# Endovascular Repositioning of a Central Venous Port Malposition in the Internal Thoracic Vein

**DOI:** 10.5334/jbsr.2231

**Published:** 2021-01-11

**Authors:** Mike Salavracos, Fabrice C. Deprez

**Affiliations:** 1 UCL St-Luc Bruxelles, BE; 2 CHU UCL Namur, BE

**Keywords:** Port, Malposition, Complication, Thrombosis, Endovascular

## Abstract

Malpositioning of a central venous port in the internal thoracic vein can be difficult to check based on single-plane (PA) chest radiographs only, and can be managed by interventional radiology.

**Teaching Point:** Central venous port malposition in the internal thoracic vein must be detected on postero-anterior chest radiograph and can be repositioned via endovascular procedure.

## CASE HISTORY

We report the case of a 48-year-old woman recently diagnosed with Hodgkin lymphoma. A central venous port (CVP) was inserted in the left sub-clavicular vein by surgery, with no reported complication. A frontal chest radiograph to check the catheter’s position was wrongfully interpreted as normal, but actually shows an abnormal angulation of the CVP device at the opening of the brachiocephalic vein in the superior vena cava (***Figure A***, arrow).

**Figure A:**
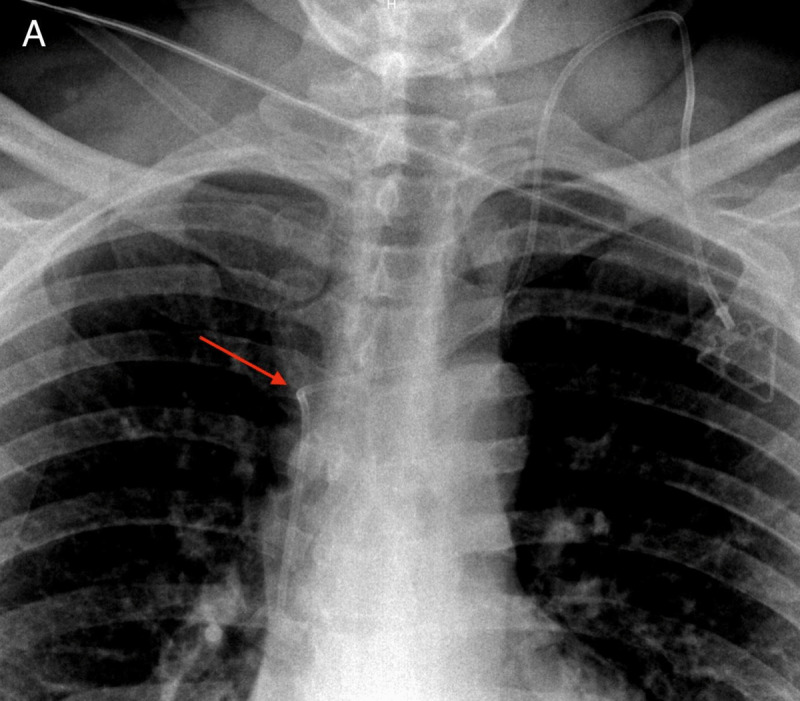


No problem was reported when the first chemotherapies were administered via the CVP. After several chemotherapies, the patient started to complain of chest pain on the right side. Moreover there was no more blood reflux.

After re-assessment of the initial chest radiograph, a C-arm cone-beam computed tomography was ordered and demonstrated the incorrect anterior positioning of the last centimeters of the catheter, presumably in the right internal thoracic vein (arrow ***Figure B***). Opacification by iodine contrast injection through the CVP showed thrombosis around the tip of the catheter with backflow to the brachiocephalic vein before antegrade flow (arrows, ***Figure C***, the white dotted line shows the catheter through ostium of the right internal thoracic vein). Via femoral vein access, we created a loop around the CVP catheter in the left brachiocephalic vein with a guidewire and a snare, allowing for removal of the catheter by traction from the internal thoracic vein.

**Figure B:**
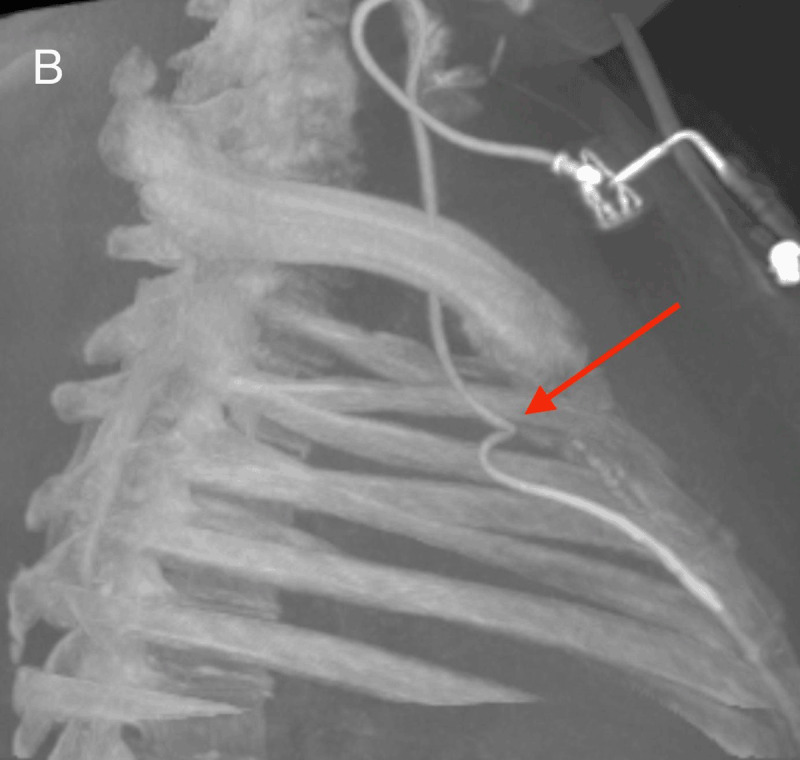


**Figure C:**
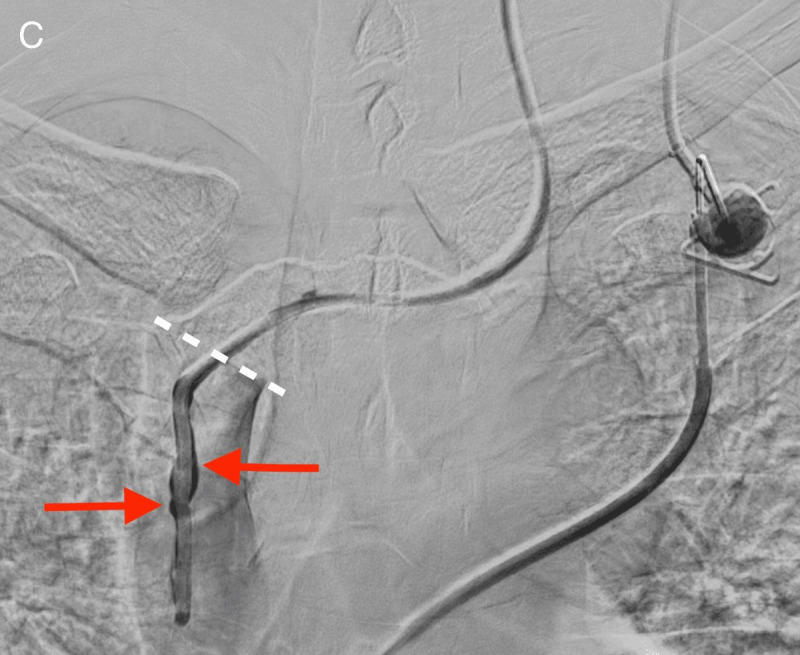


## COMMENTS

The most common complication relating to intravenous devices within the first 30 days after placement is an inadequate positioning of the catheter, whereas later complications include infections, catheter fracture, and thrombosis or stenosis [1].

Radiographic controls are effective in detecting incorrect positioning, fracture, or migration of the catheter. Pain or resistance during injection or absence of blood reflux must lead to CVP revision with iodine contrast injection.

Most of the catheter-related complications can be managed via endovascular techniques from a jugular or femoral vein access, especially catheter misplacement as in the present case. When the tip of the CVP is reachable, it can be directly hooked by a single- or multi-loops endovascular snare and pull or push in the right position; if not, such as in this case, a loop can be created around the CVP catheter with a guidewire and a snare.
